# The Influence of the Joint Volume on the Prevention of Impingement and Elbow-at-Side Rotations: Could the 36 mm Sphere with an Inferior Offset of 2 mm Be the New Gold Standard?

**DOI:** 10.3390/jcm14072324

**Published:** 2025-03-28

**Authors:** Marion Besnard, Ramy Samargandi, Osamah Abualross, Julien Berhouet

**Affiliations:** 1 Centre Hospitalier Intercommunal d’Amboise, Rue des Ursulines-BP 329, 37403 Amboise Cedex, France; marion.besnard22@gmail.com; 2 Department of Orthopedic Surgery, College of Medicine, University of Jeddah, Jeddah 23218, Saudi Arabia; rsamargandi@uj.edu.sa; 3 College of Medicine, University of Jeddah, Jeddah 23218, Saudi Arabia; osamahhanisaleh@gmail.com; 4 Service de Chirurgie Orthopédique et Traumatologique, Centre Hospitalier Régional Universitaire (CHRU) de Tours, 1C Avenue de la République, 37170 Chambray-les-Tours, France

**Keywords:** reverse shoulder arthroplasty, scapular impingement, implant design, notching, preoperative planning, joint volume

## Abstract

**Background**: Reverse shoulder arthroplasty (RSA) improves shoulder function in cases of glenohumeral osteoarthritis and rotator cuff arthropathy. The design of the glenosphere influences mobility and scapular impingement. This study evaluates the impact of joint volume on the range of motion (RoM) and identifies design modifications to enhance mobility while reducing the impingement risk. **Methods**: Thirty-four cadaveric shoulders were implanted with the Aequalis Reversed II^®^ prosthesis in seven configurations: four with 36 mm spheres (centered, 2 mm eccentric, and lateralized by 5 mm and 7 mm) and three with 42 mm spheres (centered, and lateralized by 7 mm and 10 mm). The joint volumes (inferior, anteroinferior, and posteroinferior) were measured via 3D CT scans. The RoM in adduction and elbow-at-side rotations (IR1 and ER1) was recorded. A statistical analysis identified threshold joint volumes correlating with improved mobility. **Results**: Larger joint volumes correlated with enhanced mobility. The 42 mm spheres demonstrated better adduction and ER1 compared to those of the 36 mm spheres (*p* < 0.0001). An inferior volume > 5000 mm^3^ and anteroinferior/posteroinferior volumes >2500 mm^3^ were thresholds for significant mobility improvement. Lateralization (≥7 mm) or inferior eccentricity (2 mm) improved the mobility with the 36 mm spheres, with the 36 + 2 configuration offering a practical balance for smaller patients. **Conclusions**: Increased joint volume enhances mobility, particularly in adduction and elbow-at-side rotations. A sphere with a 2 mm inferior offset or a 42 sphere with 7 mm lateralization optimizes the RoM while minimizing impingement risks. Patient-specific considerations, including anatomy and soft tissue tension, remain essential for optimal prosthesis selection.

## 1. Introduction

The reverse shoulder arthroplasty (RSA) was introduced by Paul Grammont [1] nearly 40 years ago for the treatment of glenohumeral osteoarthritis caused by rotator cuff arthropathies. By medializing and moving the center of rotation of the glenohumeral joint inferiorly, the RSA enhances the lever arm of the deltoid muscle, which then becomes the main motor of the shoulder. The excellent functional performance of this prosthesis has resulted in its widespread use and an expansion of its indications, which include irreparable massive cuff tear arthropathy, pseudoparalysis, proximal humerus fractures in the elderly, and fracture sequelae [2,3,4,5].

A number of modifications to the Grammont RSA have been suggested and it is now acknowledged that inferior glenosphere protrusion and lateralization, whether bony or metallic, reduce the risk of scapular notching [6,7,8,9,10].

The postoperative internal rotation (IR) in RSA is difficult to predict, although some factors favoring recovery have been identified [11,12,13,14,15,16]. Rol et al. showed that lateralization from the glenoid and an increase in the diameter of the glenosphere were significantly associated with a better postoperative IR [11]. These modifications enhance the inferior joint volume, which suggests that the limitation of the postoperative IR may be related to a reduced available volume beneath the glenoid component. The inferior joint volume plays a pivotal role in the biomechanics of the RSA. An adequate inferior volume reduces the risk of impingement by increasing the available space for movement, particularly during adduction and IR [11]. Additionally, a previous study on anatomic specimens demonstrated that shoulder mobility is influenced by the anatomy of the scapular pillar, increasing with the increase in the area under the pillar. Notably, this research was conducted in a two-dimensional (2D) framework [17].

With the widespread use of three-dimensional (3D), planning software [18,19,20], we aimed to evaluate the influence of the joint volume beneath the scapular pillar on the range of motion (RoM), and more specifically, the distribution of this ‘functional volume’ in the axial plane, thereby allowing for a more precise definition of the impingement zones between the prosthesis and the scapular pillar.

The aim of this study was to analyze the inferior joint volume based on the type of glenoid sphere and to correlate these results with the RoM. The working hypothesis was that increasing the joint volume beneath the scapular pillar would improve the RoM, particularly for rotations and adduction. Additionally, this study seeks to elucidate how variations in the inferior joint volume affect the incidence of impingement, ultimately guiding implant design for better clinical outcomes.

## 2. Materials and Methods

In total, 34 cadaveric shoulders were used in this study, from an initial database of 40 specimens (20 right, 20 left). These shoulders were free of osteoarthritis or fracture sequelae, and were divided into 21 males and 19 females, with a mean age at death of 79.1 years (61–95 years). Information on donor height and weight was not available. Each shoulder corresponded to the entire upper limb, including scapula, humerus, clavicle, forearm, and hand.

For each shoulder, the Aequalis Reversed II^®^ prosthesis (Tornier Inc., Edina, MN, USA) was implanted, with an Inlay-type humeral stem with 155° angulation fitted with a standard +6 mm insert, a 25 or 29 mm diameter glenoid plate, and a glenosphere in 7 different configurations:

- For the 36 mm diameter sphere, the following were used:
  - Standard center: 36 + 0.
  - Eccentric with 2 mm lower offset: 36 + 2.
  - 5 mm lateralized (equivalent to a BIO (Biologic Increased Offset) or a MIO (Metallic Increased Offset)—RSA (Reverse Shoulder Arthroplasty): 36 + 5.
  - 7 mm lateralized: 36 + 7.
- For the 42 mm diameter sphere, the following were used:
  - Standard center: 42 + 0.
  - Offset by 7 mm: 42 + 7.
  - Offset by 10 mm: 42 + 10.

### 2.1. Implantation of the Prosthesis and Measurement of Joint Mobility

RSA was performed following a detailed and reproducible protocol by a single surgeon (JB). A Sawbones^®^ modular metal rack (Malmö, Sweden) was used. It comprised a clamp to fix the scapula and an articulated arm to which the remaining upper limb was attached by means of a centromedullary pin screwed to the humeral implant. The assembly replicated the various movements of the shoulder, while allowing amplitude measurements to be taken using protractors in the frontal and horizontal planes. The purpose of the assembly was to be able to modify the elevation of the humerus while allowing rotational measurements.

On the glenoid side, a 25 or 29 mm diameter baseplate was associated with a 36 or 42 mm diameter sphere, after preparation of the glenoid around a threaded guide pin positioned perpendicular to the joint surface and according to the 12 mm rule reported by Kelly [21]. On the humeral side, a 6.5 mm diameter stem with a metaphysis of a diameter adapted to the implanted glenosphere, combined with an insert of the same diameter, was used. Retroversion of the humerus was adjusted using a pin positioned in relation to the axis of the forearm.

**Figure 1:**
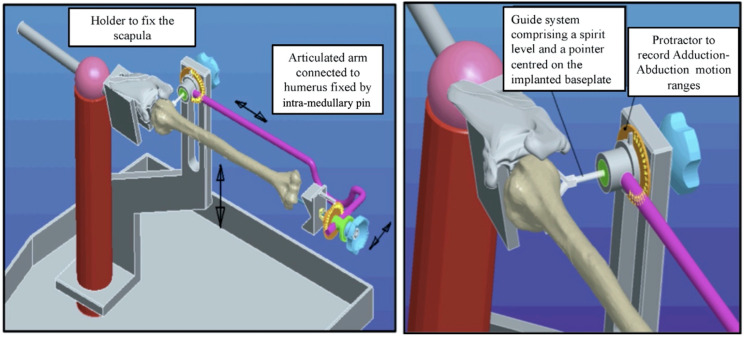
An articulated metal rack allowing the shoulder to move in all planes and a system used to place the prosthetic glenoid in the zero-reference position (the forearm and hand are not shown on the drawings but were present on the cadaveric subjects).

**Figure 2:**
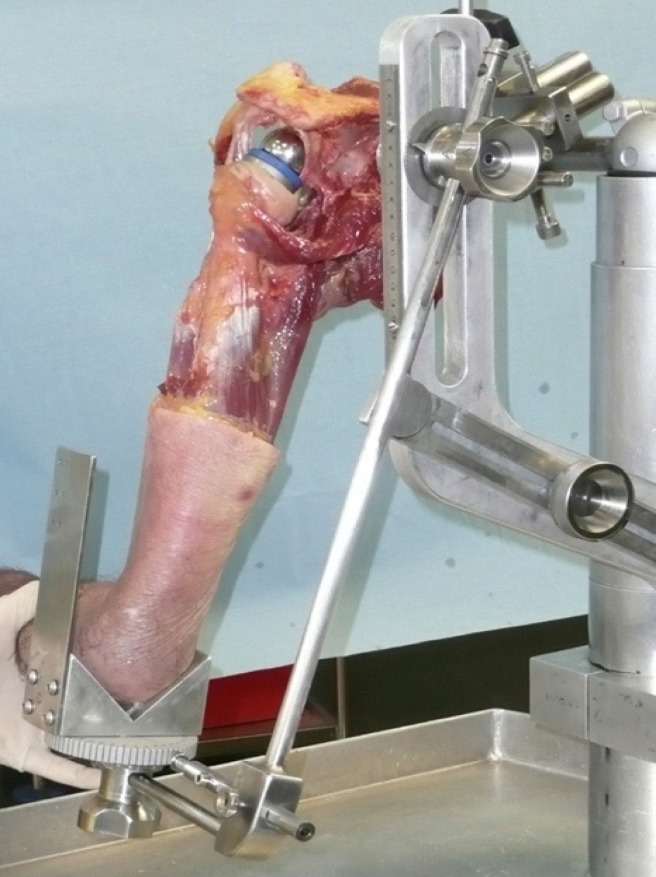
Final assembly of the cadaveric shoulder, into which a reverse shoulder arthroplasty has been implanted, placed in an articulated metal rack, before measurements.

After preparation of the shoulder prosthesis’ assembly on the articulated metal frame, the various measurements were taken following an essential step to adjust the plumbness of the glenoid, in a bid to ensure that the center of rotation of the glenohumeral joint was in the same axis as that of the metal frame (Figure 1 and Figure 2). The reference plane was then that of the scapula, meaning that rotation was neutral when the forearm on the elbow bent at 90° was perpendicular to the plane of the scapula.

Maximum mobility in internal and external rotation with elbow at the side (IR1 and ER1, respectively) was recorded for each of the 34 implanted cadaveric shoulders. The peak amplitudes corresponded to the occurrence of inferior, anterior, and posterior impingement. The abduction level of the humerus at which rotations were assessed was 20°. This was defined on the basis of the glenometaphyseal angle, which reflects the relationship between the position of the glenoid sphere and that of the humeral implant. In the study conducted by Falaise et al. [22], the average glenometaphyseal angle was 46.9° in patients with notches, which corresponded to an abduction of the humerus of 20°, whereas the angle in patients without notches was 37.5°, corresponding to an abduction of 52°, with the glenoid strictly vertical. This low value was deliberately selected to detect rotational conflicts.

### 2.2. Data Acquisition Protocol

A CT scan (Somatome^®^ Definition AS+ machine, Siemens S.A.S., Courbevoie, France) of each shoulder was performed at the end of the experiment, using a standardized image acquisition protocol.

**Figure 3:**
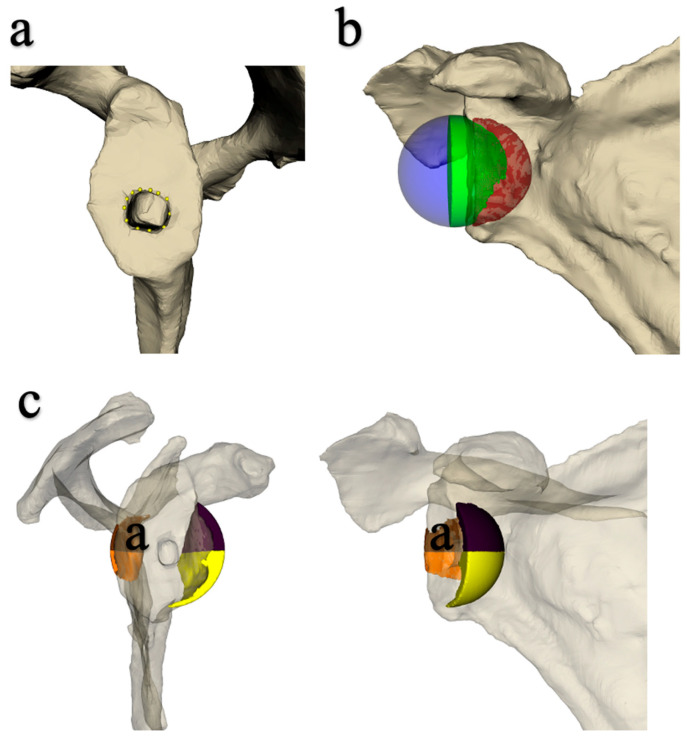
Segmentation of scan images. (**a**) Determining the entry point of the glenoid plate. (**b**) Placing the sphere: in purple: implant volume; red: joint volume with sphere +0; green: joint volume with lateralized sphere. (**c**) Segmentation of the joint volume into 3 parts: “face-view” and frontal (coronal plane) views, with the prosthetic glenosphere removed. Yellow: anteroinferior joint volume. Orange: posteroinferior joint volume. Purple: superior joint volume.

Each scan was hand-segmented to create a scapular volume. A ‘marching cubes’ algorithm was then used to generate a 3D mesh representing the segmented scapula. Each scapula 3D mesh was manually annotated to identify the glenoid stage entry point and the plane passing through the back of the stage. The entry point of the implant was the center of the hole for the glenoid plate stud (Figure 3a). The plane passing through the back of the plate was calculated as the plane that best corresponded to the manually defined points on the glenoid (where the plate had been implanted).

The glenoid implant and joint volumes were then generated by placing the center of a sphere of the desired diameter on the entry point of the glenoid plate. For lateralized implants, the center of the sphere was moved laterally to a distance corresponding to the lateralization (5 and 7 mm). For the 36 + 2 eccentric spheres, meanwhile, the center of the sphere was displaced 2 mm inferiorly. The sphere was then cut by the plane of the glenoid into two volumes: one representing the implant itself and the other the joint volume (Figure 3b). This joint volume was then split into 4, in 2 planes perpendicular to the plane behind the glenoid plate, giving 4 parts: anteroinferior, posteroinferior, anterosuperior, and posterosuperior (Figure 3c). Logically, only the lower volumes were used to analyze the occurrence of impingement with the scapular pillar. The volumes for each part were calculated using the VTK software library (Version 9.1.0). Measurements were taken by a single observer (SP).

### 2.3. Statistical Analysis

A descriptive statistical summary was compiled for each of the quantitative joint mobility and volume variables: minimum, quartiles (Q1, median, and Q3), mean, and maximum. The gains were then calculated as a percentage from the means of each variable. There were no qualitative variables. The relationship between joint volume and mobility was analyzed using scatter plots to define a threshold value of joint volume, above which mobility was significantly different. All the statistical tests were non-parametric based on the data characteristics and study design, and the *p*-value was estimated using the Monte Carlo method (10,000 iterations). The significance threshold was set at 0.05. Mann–Whitney tests were used to compare 2 means, and Kruskal–Wallis tests were used to compare 7-sphere means. All statistical processing was carried out by a statistician (CT) using XLSTAT Life Sciences software (Addinsoft, Bordeaux, France) version 2021.5.

## 3. Results

The descriptive data are summarized in Table 1.

### 3.1. Influence of Sphere Design on Mobility in Adduction, ER1, and IR1 (Table 2, Table 3 and Table 4)

The mobility was considerably better with the 42 + 0 spheres than with the 36 + 0 spheres (*p* < 0.0001), but there was no significant difference with the other 36 spheres.

The lateralized 42 spheres showed better adduction and ER1 mobility than the 36 spheres. There was no significant difference in IR1 between the lateralized 42 spheres and the 36 + 2 spheres, or between the 42 + 7 and 36 + 7 spheres.

The adduction was significantly lower with the 36 + 0 than with the 36 + 2 spheres. The 36 + 2 and 36 + 7 spheres had significantly better rotational mobility than the 36 + 0 spheres, but there was no significant difference between the 36 + 2 and 36 + 7 spheres. With the 42 + 10 spheres, the adduction and ER1 were better than with the 42 + 0 spheres.

**Table 2:** Gains in adduction according to sphere design.

	36	36 + 2	36 + 5	36 + 7	42	42 + 7	42 + 10
36	1	**0.037**	**0.007**	**<0.0001**	**<0.0001**	**<0.0001**	**<0.0001**
36 + 2		1	1.000	0.663	0.477	**0.002**	**<0.0001**
36 + 5			1	0.682	0.615	**0.000**	**<0.0001**
36 + 7				1	1.000	**0.020**	**0.000**
42					1	0.318	**0.016**
42 + 7						1	0.800

**Table 3:** ER1 gains according to sphere design.

	36	36 + 2	36 + 5	36 + 7	42	42 + 7	42 + 10
36	1	**0.010**	0.126	**0.001**	**<0.0001**	**<0.0001**	**<0.0001**
36 + 2		1	0.993	0.997	0.751	**0.011**	**0.000**
36 + 5			1	0.661	0.139	**0.000**	**<0.0001**
36 + 7				1	0.970	**0.019**	**0.000**
42					1	0.191	**0.007**
42 + 7						1	0.902

**Table 4:** IR1 gains according to sphere design.

	36	36 + 2	36 + 5	36 + 7	42	42 + 7	42 + 10
36	1	**0.022**	0.198	**0.012**	**<0.0001**	**<0.0001**	**<0.0001**
36 + 2		1	0.976	1.000	0.868	0.191	0.099
36 + 5			1	0.997	0.321	**0.025**	**0.007**
36 + 7				1	0.653	0.059	**0.014**
42					1	0.988	0.909
42 + 7						1	1.000

### 3.2. Influence of Sphere Design on Joint Volume (Inferior, Posteroinferior and Anteroinferior)

The joint volume was significantly greater with the 42 + 0 spheres than with the 36 + 0 spheres. The further the sphere was lateralized, the greater the volume, particularly for the posteroinferior volume. In general, lateralization increased the volume more than the inferior eccentricity (Table 5 and Table 6).

### 3.3. Correlation Between Joint Volume and Mobility

**Figure 4:**
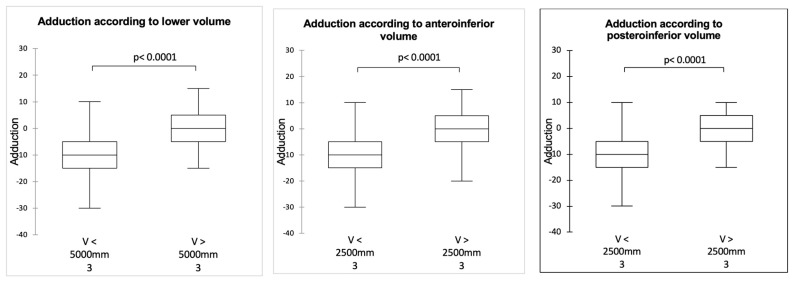
Adduction according to joint volume.

**Figure 5:**
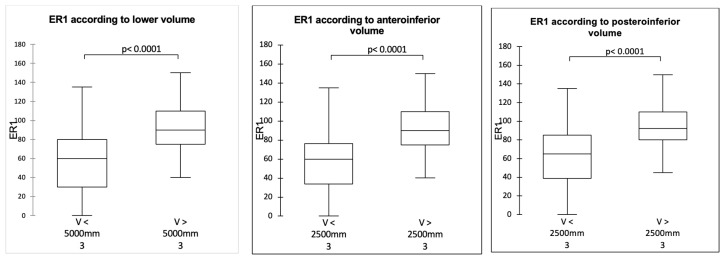
ER1 according to joint volume.

**Figure 6:**
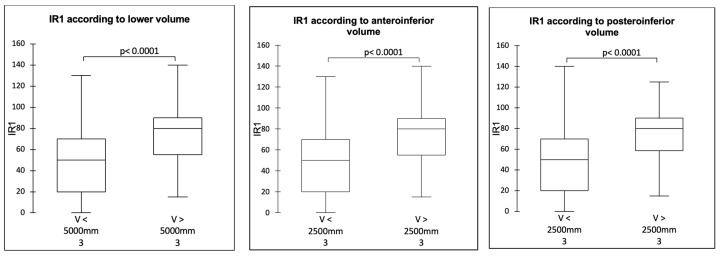
IR1 according to joint volume.

It was possible to define the threshold joint volume values above which the mobility was significantly improved: 5000 mm^3^ for the inferior volume and 2500 mm^3^ for the anteroinferior and posteroinferior volumes (*p* < 0.0001). The values established two groups: a group made up mainly of 36 mm spheres whose volumes were below the thresholds and a group of 42 mm spheres whose volumes were above the thresholds (Figure 4, Figure 5 and Figure 6).

## 4. Discussion

The most important findings of our study were that larger joint volumes correlated with improved shoulder mobility, with a 36-sphere featuring a 2 mm inferior offset or a 42 mm sphere with a 7 mm lateralization optimizing mobility and supporting our hypothesis. Additionally, the results emphasize that the inferior joint volume is critical in preventing impingement, as an increased joint volume, particularly inferiorly, allows for greater freedom of movement without prosthetic contact with the scapular pillar. This highlights that a larger joint volume through prosthetic design not only enhances shoulder mobility but also significantly reduces the risk of impingement during shoulder movements.

The modification of shoulder anatomy brought about by RSA is a source of bony impingement during RoM, which may limit the recovery of movement [23,24,25]. Impingement of the acromion limits mobility in abduction and anterior elevation, while impingement of the scapular pillar limits elbow-at-side mobility [26,27,28]. Such impingement reflects a problem of available space around the prosthesis, particularly under the scapular pillar, as shown by Berhouet et al. [17]. So-called “favorable” morphologies of the scapula have been identified: an angle of the scapula [27] of less than 105° and an area under the scapular pillar greater than 0.8 cm^2^ would allow significantly better rotational elbow-at-side mobility. Nevertheless, these observations were based on two-dimensional CT scans. The current study, this time three-dimensional, shows that the greater inferior joint volume achieved by increasing the size of the glenosphere is correlated with better adduction and rotation mobility, with differences exceeding the minimum clinically significant difference [29]. This effect became significant above a 5000 mm^3^ inferior volume and 2500 mm^3^ anteroinferior or posteroinferior volume, which corresponded to the situation of implanting a 42 mm sphere, respectively, without and with lateralization.

However, increasing the diameter of the glenosphere is not the only technique to avoid impingement. Different types of glenoid implant design are now available: lateralized (in the sphere or plate), off-center, or even inferiorly inclined. In our study, the mobility evaluated was no better with a 42 mm sphere than with a 36 mm sphere lateralized or inferiorly eccentric. An inferior eccentricity of 2 mm was sufficient to enhance the adduction and rotation RoM, whereas a significant lateralization of +7 mm or even +10 mm was typically required on a 42 mm sphere to significantly improve mobilities [30]. This finding confirms that the increase in volume under the scapular pillar, beyond a threshold value, induced by a large-diameter sphere with lateralization, is important for improving joint mobility. Nevertheless, it is also interesting to note that the functional gain of a +10 mm lateralization for a 42 mm sphere is not significantly better than that observed with a 7 mm lateralization. Huish et al. [12], in a software study, found that inferiorization and an increased glenosphere size improved IR1, in contrast to isolated 7 mm lateralization. These findings seem to concur with those found in our study. He also noted that it was the combination of these three changes in glenoid configuration that produced the best IR.

Using computer modeling, Arenas-Miquelez et al. [31] reported an improvement in the overall shoulder mobility thanks to glenoid lateralization, with no difference between +4 mm and +12 mm. However, King et al. [32] demonstrated in a retrospective case–control study that isolated glenoid lateralization (+4 mm) decreased the incidence of scapular notching but did not improve the RoM. This was an in vivo study that considered the scapulothoracic and soft tissues, which may explain the differences with cadaveric studies and computer modeling. The alternative explanation could be insufficient lateralization to show an improvement in the RoM. As reported previously, a lateralization of +10 mm with a sphere of 42 mm did not significantly improve the joint mobility obtained with a lateralization of +7 mm, despite an increase in the volume under the neck of the scapula, both anteriorly and posteriorly. This might be an argument for saying that there is probably a “threshold effect for functional volume” necessary for good mobility. This finding reinforces the fact that the problem of impingement and elbow-at-side rotation after RSA is not just a question of osteo-prosthetic relationships, but also of the surrounding soft tissues. Although the present study was carried out on cadavers and was therefore ‘passive’, it probably serves to illustrate the limitation provided by the soft tissues, through excessive tension and thus a hindrance to mobility, depending on the configurations of the implants tested. Such an analysis has already been carried out by Rol et al. [11], as well as by Berhouet et al. [33] in clinical studies, both of which found a better IR1 magnitude when the teres minor was absent or atrophic.

Similarly to our study, other studies in the literature evaluating the influence of different prosthesis configurations on the postoperative RoM are primarily based on cadaveric models or computer simulations [12,26,31,34,35]. The absence of consideration of the scapulothoracic joint for movement analysis is a real limitation. It has been reported that actual mobilities differ from those obtained by computer simulation, with motions in anterior elevation and abduction largely underestimated by these software programs, partly because the scapulothoracic joint is not taken into account. However, there was no significant difference for the ER1, as the scapulothoracic joint plays a small role in the ER1 [18,36]. The lack of soft tissue consideration is a limitation of our study; however, as previously reported, indirect soft tissue involvement cannot be ruled out when assessing passive mobility in cadaveric models. Notwithstanding the limitations of cadaveric models, they appear to be more realistic than computer simulators, especially in the case of whole specimens with preserved soft tissue, as we were able to use in our study.

Based on the findings of this study, we can recommend that the centered 36 mm sphere should no longer be used, and that a 36 mm sphere eccentric by 2 mm should be used instead. The latter remains easy to use, even in smaller-sized patients. It guarantees an increase in rotational mobility, while limiting the notching compared to a centered implant, even with a larger diameter (42 mm). The alignment of the 2 mm inferior overhang with the scapular pillar should further increase the joint volume and thus improve the prevention of inferior impingement and mobility. The use of a 42 mm sphere, however, is reduced. If the patient’s size allows it, an additional lateralization of 7 (rather than 10 mm) is sufficient to provide a significant functional gain. An intriguing compromise, already mentioned in a previous study but not clearly evaluated until now, would be to use a sphere centered at 39 mm, or even eccentrically inferior by 2 mm, thus allowing a theoretical functional gain while limiting excessive stress on the soft tissues.

The selection of the RSA configuration must therefore take all of these parameters into account, in addition to the consensus recommendations allowing for an increase in the inferior joint volume, in order to improve mobility and avoid conflict with the scapular pillar.

The strengths of this study include the use of 34 cadaveric shoulders, which allowed for a controlled analysis of various prosthesis configurations, and the utilization of 3D CT imaging to accurately measure joint volumes and their correlation with mobility. Additionally, this study provides clinically relevant recommendations for implant selection to optimize postoperative shoulder function. However, limitations include the absence of soft tissue consideration in cadaveric models, which may not fully replicate in vivo conditions, the lack of patient-specific anatomical data such as height and weight, and the inherent limitation that cadaveric studies do not account for postoperative rehabilitation, soft tissue healing, or long-term clinical outcomes in real patients. Another limitation is the potential variability introduced by cadaveric models themselves, including differences in preservation, tissue integrity, and anatomical variation, which can affect joint volume measurements and mobility assessments. Future studies should address these factors for a more comprehensive analysis.

Future advancements in preoperative planning and implant design hold potential for refining these principles further. The integration of artificial intelligence-driven modeling and motion simulation software may enhance the precision of patient-specific surgical planning, allowing for optimized implant positioning to maximize mobility while minimizing complications. Moreover, the application of 3D printing technology in preoperative simulations and the development of patient-specific implants present promising avenues for improving surgical accuracy and personalization of treatment. These innovations may contribute to enhanced functional outcomes and increased implant longevity in reverse shoulder arthroplasty.

## 5. Conclusions

The increase in the inferior joint volume, primarily by inferior eccentricity or by lateralization of +7 mm for a 36 mm sphere, as well as by increasing the diameter to 42 mm, with or without a lateralization of 7 mm to 10 mm, makes it possible to increase mobility in adduction and internal and external elbow-at-side rotation. It remains essential, however, to consider the soft tissue and morphology of the patient, in order to prevent the undesirable effects of an excessive increase in periprosthetic stress (pain and spine fracture).

## Figures and Tables

**Table 1:** Descriptive data.

Sphere	Supply (°)	ER1 (°)	IR1(°)	Lower Volume (mm’)	Anteroinferior Volume (mm’)	Posteroinferior Volume (mm’)
36	−16.5 [−35–5]	32.4 [0–125]	28.2 [0–100]	2419.9 [1349.2–4091.6]	1444.9 [342.5–2260.7]	973.6 [89.4–1957]
36 + 2	−9.3 [−30–15]	64.6 [0–135]	55.9 [0–130]	2855.6 [1580.5–4451.8]	1713.0 [435.5–2369.9]	1138.7 [299–2126.3]
36 + 5	−9.1 [−20–5]	55.9 [0–135]	48.2 [0–100]	3465.6 [2589.5–4552.1]	1866.4 [1301.7–2408.9]	1597.6 [946.8–2189.9]
36 + 7	−6.3 [−20–10]	70.4 [0–130]	54.7 [0–105]	3980.7 [3264.8–4797.5]	2078.2 [1762.7–2507.4]	1899.5 [1402.4–2326.8]
42	−5.1 [−25–15]	77.2 [−0–150]	67.6 [0–140]	5453.5 [3907.7–7527.3]	3102.0 [1636.2–4143.3]	2346.6 [1075.6–3652.6]
42 + 7	−0.7 [−15–10]	94.1 [45–145]	74.7 [15–120]	6850.0 [5497.7–7961]	3619.0 [2759.7–4352.6]	3225.5 [2218.7–3826.1]
42 + 10	1.8 [−15–10]	101.0 [55–145]	77.5 [20–125]	7606.3 [6518.6–8505.8]	3923.8 [3416.5–4494.5]	3678.3 [2929.7–4175.6]

**Table 5:** Volume gains according to sphere design: compared with the 36 + 0 mm sphere.

	36 + 2 mm Sphere	36 + 5 mm Sphere	36 + 7 mm Sphere	42 + 0 Sphere
Inferior volume	18%	43%	65%	125%
Anteroinferior volume	19%	29%	44%	115%
Posteroinferior volume	17%	64%	95%	141%

**Table 6:** Volume gains according to sphere design: compared with 42 + 0 sphere.

	42 + 7 Sphere	42 + 10 Sphere
Inferior volume	26%	39%
Anteroinferior volume	17%	26%
Posteroinferior volume	37%	57%

## Data Availability

The data presented in this study are available upon request from the corresponding author.
